# Working in preventive medicine or not? Flawed perceptions decrease chance of retaining students for the profession

**DOI:** 10.1186/s12960-019-0368-2

**Published:** 2019-05-15

**Authors:** Van Anh Thi Nguyen, Karen D. Könings, E. Pamela Wright, Hoat Ngoc Luu, Albert J. J. A. Scherpbier, Jeroen J. G. van Merriënboer

**Affiliations:** 1 Department of Medical Education and Skills Laboratory, Hanoi Medical University, 1 Ton That Tung street, Dongda, Hanoi, Vietnam; 2 Faculty of Health, Medicine and Life Sciences, School of Health Professions Education (SHE), Maastricht University, P.O. Box 616, 6200 MD Maastricht, the Netherlands; 3 Guelph International Health Consulting, Frederik Hendrikstraat 18, 1052 HT Amsterdam, the Netherlands; 4 Biostatistics and Medical Informatics Department, Institute for Preventive Medicine and Public Health, Hanoi Medical University, 1 Ton That Tung street, Dongda, Hanoi, Vietnam

**Keywords:** Student perception, Characteristics of specialty, Preventive medicine, First-choice students, Student motivation, Career choice

## Abstract

**Background:**

Recruiting and retaining students in preventive medical (PM) specialties has never been easy; one main challenge is how to select appropriate students with proper motivation. Understanding how students perceive PM practice differently from practicing doctors is necessary to guide students, especially for those for whom PM is only a substitute for medicine as their first study preference, properly during their study and, later, the practice of PM.

**Methods:**

One thousand three hundred eighty-six PM students in four Vietnamese medical schools and 101 PM doctors filled out a questionnaire about the relevance of 44 characteristics of working in PM. ANOVAs were conducted to define the relationship between students’ interest, year of study, willingness to work in PM, and the degree to which students had realistic perceptions of PM practice, compared to doctors’ perceptions.

**Results:**

Overall, compared to doctors’ perceptions, students overestimated the importance of most of the investigated PM practice’s characteristics. Moreover, students’ perception related to their preference and willing to pursue a career in PM after graduation. In particular, students for whom PM was their first choice had more realistic perceptions of community practice than those who chose PM as their second choice. And, second-choice students had more realistic perceptions than first-choice students in their final years of study, but expected higher work stress in PM practice. Students who were willing to pursue a career in PM rated the importance of community practice higher than those who were not. We also found that students’ perception changed during training as senior students had more realistic perceptions of clinical aspects and working stress than junior students, even though they overemphasized the importance of the community aspects of PM practice.

**Conclusions:**

To increase the number of students actually entering the PM field after graduation, the flawed perceptions of students about the real working environment of PM doctors should be addressed through vocation-oriented activities in the curriculum targeted on groups of students who are most likely to have unrealistic perceptions. Our findings also have implications for other less attractive primary health care specialties that experience problems with recruiting and retaining students.

## Background

Preventive medicine (PM) should be a priority in developing countries where preventable diseases cause death of millions of people every year, especially of children under 5 years old [1]. However, it is an international problem that primary health care (PHC) specialties, such as PM, are not attractive to medical students [2, 3]. Previous studies have shown that students’ knowledge and experiences of specialties influence their career decision-making processes [4]; they increase or decrease students’ preferences for PHC careers [5, 6] and working in rural settings [7]. Insufficient understanding or knowledge of the role of PHC specialties is a major factor discouraging students from pursuing a career in those specialties [8, 9]. However, little is known about the development of students’ perceptions of PHC specialties over time. Particularly, it is underexplored how the perceptions of students with different specialization interests develop in different phases of the curriculum, and whether students’ perceptions of the specialty relate to their willingness to work as a PHC doctor.

Medical students gradually collect more knowledge and experiences during different phases of their studies, mostly during their training in clinical clerkship [10, 11] and community practice [7]. Based on those experiences, senior students make more definite and consistent decisions on their career choice in PHC than junior students do [9]. Nonetheless, some studies show that the career preferences of students are not always related to their actual knowledge of the specialty [8, 12] but primarily to their values and goals [13]. Some scholars recommend that students should be more guided to gain a more accurate view of PHC specialties [12] and that educators should consider the risk of fostering a negative attitude toward PHC specialties among students during their training [13, 14].

In medical education, motivation for or interest level in pursuing a career in a particular specialty has been found to influence medical students’ persistence in their study [15] and their specialty choice [16]. Moreover, motivation is a predictor of students’ satisfaction during the study, including levels of distress or burnout [17, 18]. Another study [19] showed that “second-choice students,” who chose to study the specialty because they were not accepted into their preferred program (i.e., general medicine), regretted their choice and wanted to change to another specialty or even another profession, more than “first-choice students,” who were admitted to their preferred program, did. A primary reason for these feelings of regret was that students were not properly informed about the specialty they chose to pursue, meaning they were not fully aware of the mission, working conditions, and job prospects. This observation is consistent with other studies noting that the major impediment to students’ career decisions is a poor understanding of the specialties [9]. However, we have not found pre-existing studies on how perceptions of a specialty vary among students who have different levels of interest in that specialty. Also, it is as yet unclear how students’ perceptions change over the course of their studies, as a result of the accumulation of experience and knowledge, and whether this impacts first-choice students and second-choice students differently. This kind of information is crucial for teachers and vocational educators who have to aid all students in forming adequate understanding about a specialty.

Studies reveal that although medical students choose to study medicine because of their desire to help people and they are well aware of the importance of PHC in the health care system, these motivations do not translate into a willingness to work in the community and in rural areas [8, 9, 12]. However, few studies have focused on the perceptions of students who have already expressed a willingness to work in the PHC field. It would be valuable to get insight into the perceptions of those studying PHC at the undergraduate level in relation to their levels of interest in PHC and their willingness to work as a PHC doctor in the future.

The fact that PHC specialties are not attractive to medical students is an international problem and can also be observed in Vietnam, where medical schools are not highly successful in recruiting appropriate PM students. Students often choose to study PM for various reasons, such as curiosity about the profession, likely high-income jobs, low entry requirements and study burden compared to general medicine, and chance to uphold family traditions [19]. The fact that none of these reasons relates to a personal passion for prevention indicates that the students may not fully understand PM, public health (PH), and the role of PM doctors in community health protection. As a consequence, it is not surprising that only 60% of PM students anticipate choosing a job within the specialty after graduation, because the rest look for a “higher prestige” job, e.g., a clinical doctor in a hospital in big cities [19]. These views seem very removed from reality where one of the main motivating factors that keep PH workers, including PM doctors, in their jobs is that they realize that their contribution to health protection is appreciated by colleagues and the community [20]. Therefore, the differences between students’ perceptions and those of doctors regarding the characteristics of PM practice should be examined in more depth.

In this study, we investigated PM students’ sense of the reality of the PM specialty by comparing their perceptions with the perceptions of practicing PM doctors, focusing on key characteristics as necessary knowledge and skills for PM doctors, day-to-day PM doctor work life, types of practice, and the pressure and benefits of work as a PM doctor. We were guided by the following research questions:

1. How realistically do students perceive the characteristics of the practice of a PM doctor?
2. How do students’ realistic perceptions change over the course of the curriculum as they acquire more experience in PM? Does degree of interest in PM affect students’ realistic perceptions at different levels in the curriculum?
3. How does the willingness to work in PM after graduation affect the realistic perceptions of students? Does willingness to work in PM after graduation affect students’ realistic perceptions, and is this impacted by degree of interest in PM?

## Methods

### Setting and participants

PM training programs in Vietnam are conducted at the undergraduate level. At the moment, this study was conducted, 6 out of 13 medical Vietnamese universities offered PM training. They recruit students who are either high school graduates for a 6-year track or those already holding a bachelor of science (nurses, public health, medical technique, etc.) for a 4-year track. The regular curriculum lasts for 6 years, with the first 2 years focusing on basic sciences and basic medical knowledge and skills, the next 2 years on clinical clerkship, and the last 2 years on knowledge and skills related to the PM specialty, while the 4-year track only contains the last 4 years [21]. Graduates obtain the Degree of Doctor of PM and can officially work as PM doctors in the community or PM centers at different levels of the health care system (national, provincial, district, commune), in hospitals (if they take post-graduate training course in clinical specialties), research institutes, non-government organizations, or in medical universities/schools [22]. In Vietnam, the preventive medical system includes public health, family medicine, and PM. While bachelors of public health work only in prevention, family medical doctors take care of health of people in a family [23]. PM doctors in Vietnam have to cover both preventive and curative duties; they treat common diseases and have to detect and prevent epidemics in the community as well [22]. This requires them to work mainly in the community or in PM centers and to serve a broad range of people.

The current study is focused on the 6-year track PM training and involved 1386 PM students from four medical universities in the North of Vietnam and 101 practicing PM doctors working in Hanoi, the capital. All participants were invited to voluntarily participate immediately after attending lectures (students) or meetings (doctors). Signed consent was acquired after the researchers explained that voluntary and anonymity of the participants was guaranteed. After participants filled out the questionnaire (taking approximately 10 min), they received a small financial compensation for their devoted time, which is ordinary in Vietnamese context. Ethical approval for the study was given by the Institutional Review Board of the Hanoi Medical University (Decision No 174/HMU-IRB) and of the other three universities’ Scientific and Technical Committees.

### Materials

A written questionnaire was developed based on earlier studies on students’ perceptions of the medical profession [12, 24]. The original questionnaire, which included 47 characteristics of the medical profession, was used in a study by Soethout [12]. In our study, in light of suggestions from practicing PM doctors in the pilot phase, three items were added: “Skill with using computers,” “Capacity to use a foreign language,” and “Work that requires a lot of travelling.” Participants were asked to indicate to which degree these characteristics applied to a PM doctor’s daily practice with the phrase: “To what extent does this characteristic apply to the profession of PM?” using a 3-point Likert scale (1 = not applicable, 2 = moderately applicable, 3 = highly applicable). Exploratory factor analysis was used to identify factors within the 50 items. This procedure resulted in seven factors that were interpreted as seven subscales, and Cronbach’s alpha was calculated for each subscale. Cronbach’s alpha of every subscale was improved by deleting items that worsened the internal consistency of that subscale. Table 1 presents the content and internal consistency reliability of the seven subscales, with Cronbach’s alphas of each subscale, separately for practicing doctors and of students. The Cronbach’s alpha values for the two subscales “Clinical practicing” (.58) and “PM working stress” (.59) were quite low, (< .60); we decided to keep these subscales because they are important characteristics of working in PM and Cronbach’s alpha of 0.6 was considered acceptable for new subscales [25]. We will interpret these results with caution. The subscale of “PM working benefits” was taken out because of too low alpha, to avoid negative effect on the study’s accuracy. This procedure produced a final questionnaire containing six subscales with 41 items.

**Table 1:** Modification of the study instrument and internal consistency reliability of the subscales

Original instrument 4 subscales—47 items	Modified instrument 7 subscales—44 items	Cronbach’s *α* (doctors)	Cronbach’s *α* (students)
1. Knowledge and skills (13 items)	1. PM knowledge and skills (11 items) (e.g., Epidemiology, Health promotion)	.75	.85
	2. Basic sciences (4 items) (e.g., Anatomy, Chemistry, Pharmacotherapy)	.67	.66
2. Nature of medical-professional practice (7 items)	3. Clinical practice (7 items) (e.g., Referral, Diagnostic skills, Treatment of patients)	.58	.59
3. Types of patients, contacts, and complaints (10 items)	4. Community practice (9 items) (e.g., Simple complaints, Long relationships with patients)	.84	.75
4. Characteristics of the daily work (17 items)	5. Daily work characteristics (5 items) (e.g., Move from place to place, Diversity of work)	.66	.74
	6. PM working stress (5 items) (e.g., Stressful work, Long working days, Routine work)	.56	.64
	7. PM working benefits* (3 items) (e.g., High income, High prestige)	.13	.40

*This subscale was taken out due to low Cronbach’s alpha for both doctors and students

Mean scores of the six subscales were computed. As data were normally distributed, parametric tests were used. The differences between the original and the modified questionnaire are presented in Table 1 as well.

### Data analysis

To answer the first research question, differences between students’ perceptions and the corresponding doctors’ answers were tested for each subscale by using independent sample *t*-tests. The value of the difference score between students’ perceptions and doctors’ perceptions will be called students’ *realistic perceptions* (i.e., a positive score indicates that students perceive the importance of a characteristic to be greater than doctors do, while a negative score indicates students’ underestimation of the importance of a characteristic as compared with doctors)*.* Effect sizes in terms of Cohen’s *d* were calculated in all analyses, in which *d* values of .2 to .3 are considered small effects, those around .5 medium effects, and larger than .8 significant effects [26]. Regarding the second and third research questions, ANOVAs were conducted to test for the impact of levels in the curriculum, willingness to work in PM, and levels of interest in PM on students’ realistic perceptions. Students were divided into the following categories: first-choice students and second-choice students; junior students from first to fourth year, and senior students in fifth and sixth year; and students willing and unwilling to work in PM. Results were presented in tables for significant effects; graphs were plotted to illustrate the interaction effects. Results for research questions 2 and 3 were considered statistically significant if the one-tailed *p* was ≤ .05. For research question 1, two-tailed tests were conducted. Data were analyzed using IBM SPSS Statistics (Version 20.0).

## Results

### General information of participants

Among PM students, there were 937 (67.60%) first-choice students, 432 (31.16%) second-choice students, and 17 students (1.22%) who did not remember their initial priority choice and therefore were excluded from the analysis. There were 513 (37.02%) senior students, and 821 (59.23%) students who stated that they are willing to work in PM after graduation. The mean age of the students was 21.57 years (*SD* = 2.24), with 61% being women. PM doctors had a mean age of 40.38 years (*SD* = 8.62; 25 to 60 years old), with 52.47% being women, and the mean length of time working in the PM field was 11.72 years (*SD* = 8.13; 1 to 32 years).

### Students’ perceptions of the characteristics of PM practice

Table 2 displays the differences in perceptions of PM practice’s characteristics between students and practicing doctors. Students significantly overestimated the characteristics of PM practice on all scales, except for clinical practice. The effect size was medium (Cohen’s *d* > .5) in most of the subscales where the differences were found significant (*p* < .05). This indicates that the differences were large enough to have practical impact, although the mean difference between students and doctors was quite low (ranging from .13 to .29).

**Table 2:** Relevance of characteristics of PM in daily work as perceived by students, compared with perceptions of practicing doctors

Subscales	PM students			PM doctors			Δ_students-doctors_		*t*	Cohen’s *d*
	*n*	*M*	*SD*	*n*	*M*	*SD*	*M*	*SD*		
PM knowledge and skills	1 386	2.91	.19	100	2.78	.23	.13	.19	7.05*	.62
Basic sciences	1 379	2.41	.38	98	2.13	.38	.26	.38	7.23*	.74
Daily work characteristics	1 377	2.92	.21	100	2.68	.33	.22	.23	10.30*	.87
Community practice	1 381	2.40	.37	100	2.15	.43	.23	.38	6.35*	.62
Clinical practice	1 380	2.37	.35	100	2.38	.41	−.01	.35	−.24	.03
Working stress	1 368	2.22	.42	100	2.10	.39	.11	.42	2.82*	.30

*Two-tailed *p* value ≤ .01

### Effects of level in the curriculum and degree of interest in PM on students’ realistic perceptions

We found a significant effect of levels in the curriculum on students’ realistic perceptions on the subscales of basic sciences (*F*(1,1360) = 4.61, *p* < .05), community practice (*F*(1,1358) = 11.36, *p* < .01), clinical practice (*F*(1,1359) = 3.55, *p* < .05), and PM working stress (*F*(1,1348) = 3.93, *p* < .05) (see Table 3). Senior students had more realistic perceptions than junior students did of basic sciences, clinical practice, and PM working stress; however, they seemed to overestimate the importance of community practice compared to junior students (for full descriptive statistics, see Appendix).

**Table 3:** Effect of level in the curriculum and interest in PM on student’s realistic perception of PM

Subscales	First-choice students (*N* = 937)				Second-choice students (*N* = 432)				Effect		
	Junior (*N* = 667)		Senior (*N* = 270)		Junior (*N* = 194)		Senior (*N* = 238)		Interest in PM	Level in the curriculum	Interaction effect
	*M*	*SD*	*M*	*SD*	*M*	*SD*	*M*	*SD*	*F*	*F*	*F*
PM knowledge and skills	.13	.20	.15	.17	.16	.10	.13	.19	.33	.09	6.07**
Basic sciences	.30	.36	.22	.37	.31	.37	.29	.42	2.67*	4.61*	1.96
Daily work characteristics	.24	.21	.25	.18	.25	.17	.22	.25	.18	1.02	3.02*
Community practice	.22	.40	.28	.33	.23	.35	.32	.34	1.07	11.36**	.15
Clinical practice	−.02	.35	−.01	.32	−.02	.39	.05	.33	2.67*	3.55*	2.01
Working stress	.16	.44	.03	.36	.10	.41	.13	.41	.85	3.93*	11.77**

*One-tailed *p* value ≤ .05. **One-tailed *p* value ≤ .01, *df* = 3

Regarding the impact of degree of interest in PM on students’ realistic perceptions, we found a significant effect in the subscales of basic sciences (*F*(1,1360) = 2.67, *p* = .05) and clinical practice (*F*(1,1358) = 2.67, *p* = .05). First-choice students had more realistic perceptions than second-choice students did of basic sciences and of clinical practice.

**Fig. 1:**
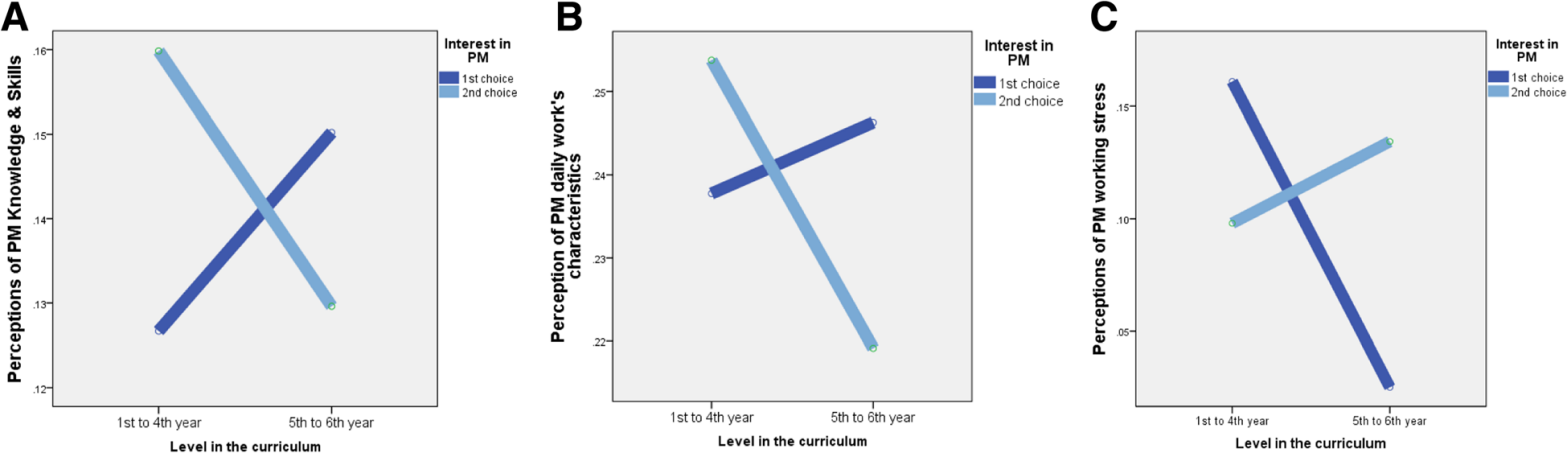
Interaction effects of level in the curriculum and interest in PM on students’ realistic perceptions of PM practice (**a**: Perceptions of PM knowlegde and skills; **b**: Perceptions of PM daily work's characteristics; **c**: Perceptions of PM working stress)

Furthermore, an interaction effect was found between the level in the curriculum and degree of interest in PM on students’ realistic perceptions of PM knowledge and skills (*F*(1,1365) = 6.07, *p* < .01), daily work characteristics (*F*(1,1356) = 3.02, *p* < .05), and working stress (*F*(1,1348) = 11.77, *p* < .01) (Fig. 1). As shown in Fig. 1a, b, first-choice students had more realistic perceptions of PM knowledge and skills and daily work characteristics at the beginning; however, they increasingly overestimated these characteristics at higher levels of study. Second-choice students gradually had more accurate perceptions of these characteristics over the time. Figure 1c shows that the more first-choice students learnt the more accurate perception of working stress they had, whereas second-choice students increasingly overestimated this when they got to their final years.

### Effect of willingness to work in PM and degree of interest in PM on students’ realistic perceptions

Regarding the effect of students’ willingness to work in PM on their perception, we found a significant effect on the subscales of PM knowledge and skills (*F*(1,1365) = 6.51, *p* < .01), daily work characteristics (*F*(1,1356) = 4.77, *p* < .05), and community practice (*F*(1,1360) = 6.73, *p* < .01) (see Table 4). Students who were willing to work in PM after graduation overestimated the importance of PM knowledge and skills, daily work characteristics, and community practice more than their peers who were unwilling to work in PM did (see Appendix).

**Table 4:** Effect of willingness to work in PM and interest in PM on student’s perception of PM

Subscales	First-choice students (*N* = 937)				Second-choice students (*N* = 432)				Effect		
	Willing (*N* = 570		Not willing (*N* = 367)		Willing (*N* = 242)		Not willing (*N* = 190)		Interest in PM	Willing to work in PM	Interaction effect
	*M*	*SD*	*M*	*SD*	*M*	*SD*	*M*	*SD*	*F*	*F*	*F*
PM knowledge and skills	.15	.16	.11	.23	.15	.15	.13	.16	1.21	6.51**	.14
Basic sciences	.29	.34	.27	.39	.28	.38	.32	.42	1.28	.01	2.39
Daily work characteristics	.24	.19	.23	.22	.25	.18	.21	.26	.27	4.77*	1.42
Community practice	.26	.36	.20	.41	.30	.33	.25	.37	4.28*	6.73**	.05
Clinical practice	−.02	.33	−.02	.36	.03	.35	.01	.37	3.58*	.03	.21
Working stress	.11	.42	.14	.43	.10	.39	.14	.43	.02	1.69	.07

*One-tailed *p* value ≤ .05. **One-tailed *p* value ≤ .01, *df* = 3

Concerning the impact of interest in PM on the realistic perceptions of students who differed in their willingness to work in PM, we found a significant effect on the subscales of community practice (*F*(1,1360) = 4.28, *p* < .05) and clinical practice (*F*(1,1359) = 3.58, *p* < .05). First-choice students, whether they were willing to work in PM or not, had more realistic perceptions than their second-choice counterparts regarding community practice and clinical practice (see Table 4). No interaction effects of willingness to work in PM and degree of interest in PM were found.

## Discussion

Our study provides new insight into the impact of levels in the curriculum and career preferences on students’ perceptions of a specific primary health care specialty, PM. The results show that students have different views than practicing doctors. More specifically, students considered most of the PM characteristics to be more important than they are in practice, even for the typical ones such as the community aspect that defines the meaning of PM, or the working stress that describe the daily job features of PM. Clinical aspect was the only one characteristic that students perceived similarly to the reality; this point was a warning signal about PM students’ tendency to pursue clinical careers. In particular, by ranking of importance of characteristics of PM based on mean subscale scores of students and doctors, we see a similar picture in the top-3 ranking. Doctors ranked the following as of most importance: (1) PM knowledge and skills, (2) daily work characteristics, and (3) clinical practice. Students chose (1) daily work characteristics, (2) PM knowledge and skills, and (3) basic sciences as their first three priorities. Although students realized the importance of daily job and the application of PM knowledge and skills in the specialty, they seemed to underestimate the application of clinical practice while they overemphasize the basic sciences in their future work. Our findings confirmed the conclusion of previous studies [8, 12, 13] that students’ career preferences are not always related to their actual knowledge of the specialty, and our results provide specific evidence about perceptions of PM from the point of view of undergraduate students. Considering the state of PM training in Vietnam, a heavily theoretical and less practical curriculum, in which the first 4 years focus on basic sciences and basic medical knowledge, could be responsible for students’ flawed perceptions of the importance of most PM aspects.

Regarding the question on how students’ realistic perceptions change over the course of the curriculum, our results show that after getting some PM experience in the fifth and sixth years, senior students developed more accurate perceptions of basic sciences, clinical aspects, and working stress, but overemphasized the importance of community in PM practice compared to the reality. Previous studies have shown that the knowledge and experience that medical students gain during practice in the field helps them to develop more realistic perceptions of a particular specialty [7, 10, 11]. Our findings confirm these observations in the case of PM, and they also indicate a notable point that training in the community should be more practical to help students to have realistic perceptions of this aspect of PM practice.

Concerning the impact of degree of interest in PM on students’ realistic perceptions, our study reveals that first-choice students always had more realistic perceptions of basic sciences and clinical practice than second-choice students, and they had more realistic perceptions of working stress than second-choice students in later years. However, second-choice students had more realistic perceptions of PM knowledge and skills and daily work characteristics than their first-choice counterparts. One explanation for this observation could be that lower interest in PM could increase the stressful feelings that second-choice students reported during their studies. Previous studies have indicated that motivation and career choice play a role in the prevalence of burnout [17, 18]. Applying this to our case, we may speculate that second-choice students, who showed a lower interest in PM and higher expectation of work stress in PM practice, could be at risk of burnout and therefore should be given more attention in efforts at prevention. Moreover, in light of previous findings about the impact of early education programs on improving attractiveness of PHC to medical students [5–7], the fact that second-choice students had more accurate perceptions of some PM characteristics in their final years is a promising sign for developing an intervention strategy. This program should early introduce the practical information such as important knowledge and skills that are useful for PM doctors, a typical work day of a PM doctor, how working in PM helps to protect the community, and so on to PM students, specifically to second-choice students who are willing to work in PM, though it was not their initial preference.

Our results revealed that students who had high willingness to work in PM overemphasized the importance of PM knowledge and skills, PM daily work characteristics, and the community aspects compared to the reality. A potential reason for this phenomenon could be that students who expressed a willingness to work in PM also had more motivation to learn and to look for information about the specialty; however, the information they found was theoretical and removed from reality. Therefore, our findings could be a foundation for developing a more appropriate information provision program, which would help students, specifically those who are not willing to work in PM, to have accurate perceptions of their future work, focusing on the role of PM knowledge and skills, PM daily work characteristics, and the community aspects of PM practice.

Remarkably, we found that first-choice students, whether they were willing to work in PM after graduation or not, had a more accurate perception of the community and clinical aspects of PM practice than second-choice students. This finding reinforces the idea of introducing community-oriented values and the concept of community interest to students during the course of training, especially to those for whom PM was not their first choice, to produce a greater number of PM doctors.

Examining the extent to which students’ perceptions of PM practice are realistic as compared to doctors’ perceptions helps us to better understand students’ specialty preferences and willingness to work in PM. Hopefully, these insights can contribute to methods of attracting more appropriate students to work in PM, by providing students with additional information during their medical studies. Our findings indicate the kinds of misconceptions students have about the real working situation of practicing PM doctors, such as their daily work characteristics or the specific knowledge and skills applied in PM practice, which could be corrected before and during studying as part of the curriculum’s vocational component. Another implication of our study is that second-choice students have a greater tendency toward stress and might be at risk of burnout. Appropriate vocational interventions focusing on community-oriented values and interests should be directed to second-choice students, especially those who are not willing to work in PM, in order to increase the number of students entering the PM field after graduation. These findings may also have implications for other less attractive PHC specialties that are struggling to recruit and retain students.

### Limitations

There are some limitations of our study. Although the original questionnaire developed by Soethout [12] used a 5-point Likert scale, we decided to use a 3-point Likert scale when asking how much a particular characteristic applied to a PM doctor’s daily practice, which could have reduced the accuracy of the scale. This option was chosen because it is general practice in Vietnam to express opinions by using one of three options: either agree, disagree, or neutral. Additionally, despite relatively low Cronbach’s alphas for the two subscales “Clinical practice” and “PM working stress,” we decided to report on these subscales because of their importance and representativeness in understanding the characteristics of PM practice. The results on these scales were interpreted with more caution. Furthermore, we recruited only PM practicing doctors who are working in a big city and may not representable for PM practice across the north of Vietnam, where we recruited part of our participating students. The different practice settings might have an effect on perception of PM doctors, and this issue deserves to be explored in more detail in further studies. Lastly, this was a cross-sectional study focusing on the perception of PM students at a particular time, and we could not provide information about the relationship between students’ perceptions of PM and their study achievements. A long-term follow-up study would be necessary to answer the question of whether the inaccurate perceptions of students affect their final career decisions and working destinations, i.e., in the community or in hospitals.

## Conclusions

PM students have a perspective on their specialty that is different from that of PM doctors. In general, they overestimate the importance of most of PM practice’s characteristics. During their studies, second-choice students report more stressful feelings about their future work than first-choice students. Although students realize the importance of PM knowledge and skills, daily work characteristics, and community practice, their perceptions are theoretical and far from reality. Identifying specific flawed points in students’ perceptions, as well as trying to encourage target subjects, such as junior students, second-choice students, and students who are not willing to work in PM, to develop a positive attitude, could be helpful for vocational interventions in the curriculum. Consequently, these measures might help to recruit and retain qualified people in the PM field.

## Appendix

**Table 5:** Descriptive statistics on realistic perceptions of PM students

Subscales	Level in the curriculum						Interest in PM						Willing to work in PM					
	Junior *N* = 873			Senior *N* = 513			First-choice *N* = 937			Second-choice *N* = 432			Willing *N* = 821			Not willing *N* = 565		
	*n*	*M*	*SD*	*n*	*M*	*SD*	*n*	*M*	*SD*	*n*	*M*	*SD*	*n*	*M*	*SD*	*n*	*M*	*SD*
PM knowledge and skills	873	.13	.18	513	.14	.19	937	.13	.19	432	.14	.16	821	.15	.16	565	.11	.22
Basic sciences	868	.30	.36	511	.25	.40	933	.28	.36	429	.30	.40	817	.29	.35	562	.27	.40
Daily work characteristics	864	.24	.20	513	.23	.22	929	.24	.20	431	.23	.22	817	.25	.19	560	.22	.24
Community practice	868	.22	.39	513	.30	.34	934	.23	.38	430	.28	.35	817	.27	.35	564	.22	.40
Clinical practice	867	−.02	.36	513	.01	.33	932	−.02	.34	431	.02	.36	819	−.01	.34	561	−.01	.37
Working stress	855	.15	.43	513	.08	.39	922	.12	.42	430	.12	.41	814	.11	.41	554	.14	.43
Working benefits	826	.87	.47	498	.84	.41	894	.87	.45	414	.82	.44	793	.85	.43	531	.87	.47

## Abbreviations

PH: Public health
PHC: Primary health care
PM: Preventive medicine
